# Social Capital and Health-Protective Behavior Intentions in an Influenza Pandemic

**DOI:** 10.1371/journal.pone.0122970

**Published:** 2015-04-15

**Authors:** Ying-Chih Chuang, Ya-Li Huang, Kuo-Chien Tseng, Chia-Hsin Yen, Lin-hui Yang

**Affiliations:** 1 School of Public Health, Taipei Medical University, Taipei City, Taiwan; 2 Department of Public Health, School of Medicine, Taipei Medical University, Taipei City, Taiwan; 3 Department of Anthropology, National Taiwan University, Taipei City, Taiwan; Alberta Provincial Laboratory for Public Health/ University of Alberta, CANADA

## Abstract

Health-protective behaviors, such as receiving a vaccine, wearing a face mask, and washing hands frequently, can reduce the risk of contracting influenza. However, little is known about how social capital may influence health-protective behavior in the general population. This study examined whether each of the social capital dimensions (bonding, bridging, and linking) contributed to the intention to adopt any of the health-protective behaviors in an influenza pandemic. The data of this study were from the 2014 Taiwan Social Change Survey. A stratified, three-stage probability proportional-to-size sampling from across the nation, was conducted to select adults aged 20 years and older (N = 1,745). Bonding social capital was measured by the frequency of neighborly contact and support. Bridging social capital was measured based on association membership. Linking social capital was measured according to general government trust and trust in the government’s capacity to counter an influenza pandemic. Binary logistic regressions were used to assess the multivariate associations between social capital and behavioral intention. The study results indicate that social capital may influence the response to influenza pandemic. Specifically, the intention to receive a vaccine and to wash hands more frequently were associated with the linking dimension and the bonding dimension of social capital, while the intention to wear a face mask was associated with all forms of social capital. The findings of this study suggest that government credibility and interpersonal networks may play a crucial role in health-protective behavior. This study provides new insights into how to improve the effectiveness of influenza prevention campaigns.

## Introduction

Since the outbreak of severe acute respiratory syndrome (SARS) in 2003, the World Health Organization (WHO) has urged countries to prepare for a possible, future influenza pandemic [1]. In June 2009, the WHO declared the first influenza pandemic, influenza A/H1N1, of the past 40 years [2]. By August of 2010, an estimated 18,449 deaths in 214 countries were due to this disease [3]. Scientists are concerned that the number of viral outbreaks will increase in the future as worldwide populations become more dense and mobile. Furthermore, habitat loss due to deforestation may cause pathogen-carrying animals to migrate closer to human settlements, which could lead to virus mutation and outbreaks of influenza pandemic [4].

During an influenza pandemic, adoption of health-protective behaviors can reduce the rate of disease transmission [5,6]. However, we know little, to date, about how people are likely to react to a pandemic crisis and how social contexts may shape a person’s intention to respond to a disease epidemic [7]. Recent studies have suggested considering the role of social capital in a person’s responses during an influenza outbreak [8,9]. People who obtain relevant health information from their interpersonal networks, the media, or their governments may decide to engage in health-protective action only if they trust that particular information source [10–13]. Some researchers have regarded the social cohesiveness and trusting relationships within a community, a county, or a country as the main components of social capital [8].

The relationship between social capital and individual health and health behavior has intrigued many researchers in the past two decades [14,15]. Three major theorists in the founding of social capital frequently cited in public health literature are Putnam, Coleman, and Bourdieu [16–18]. Putnam (1995) defined social capital as “features of social organization such as networks, norms and social trust that facilitate coordination and cooperation for mutual benefit.” He conducted research in both Italy and the United States on the relationships among social relations, civic engagement, and political and economic outcomes. He found that regions at higher levels of civic engagement, such as newspaper readership, voter turnout, and membership in various associations, had superior political and economic performance. Coleman (1988) described social capital as being imbedded in social relationships and serving as resources for people to achieve their goals. Coleman introduced various forms of social capital such as obligations, expectations, and trustworthiness that exist in social structures, information channels imbedded in social relationships, and norms and effective sanctions against deviant behavior. Bourdieu (1986), by contrast, introduced three types of capital: human capital (i.e., education), cultural capital (i.e., language), and social capital, defined as a form of group resources that accrue to individuals as a result of their membership in social networks. He suggested that social capital is often used to obtain human capital and cultural capital, which can raise a person’s social position and status in a society.

According to reviews of the social capital theories and studies, researchers have debated the number of dimensions in the concept of social capital. Szreter and Woolcock (2004) made significant efforts to categorize this concept into three dimensions: bonding, bridging, and linking social capital [19]. Bonding social capital refers to the relationships among members of a network, who see themselves as being similar (i.e., socioeconomic status). Prior studies have used the number of personal contacts with neighbors or friends to measure bonding social capital [20]. By contrast, bridging social capital refers to links across different groups that have no similar statuses or identities such as participation in clubs and organizations that represent the diversity of people. Linking social capital indicates connections to formal and institutionalized power in a society. Examples of linking social capital are voting participation and institutional trust [21,22].

Studies have suggested that possible mechanisms of social capital for the adoption of health behaviors during a disease outbreak could be via community norms promoting healthy lifestyle, diffusion of health information, promotion of access to local health services, and cohesive social networks to provide affective support [23]. Szreter and Woolcock (2004) argued that three forms of social capital are imperative to people’s health: bonding social capital in closed, interpersonal social contacts for the sharing of information; bridging social capital for the assets and information stemming from joining different types of civic association; and linking social capital for trusting relationships with governments or other civic institutions, which can result in higher compliance to recommended behaviors during an epidemic [19,24]. This study examined whether these different forms of social capital are associated with a person’s intention to adopt health-protective behaviors during an influenza pandemic. The hypothesis proposed was that each component of social capital—bonding, bridging, and linking—contributed to a person’s intent to receive a vaccine, wear a face mask, and wash hands more frequently during an outbreak of influenza pandemic.

## Methods

### Data

The data were collected from the 2014 Taiwan Social Change Survey [25]. A stratified, three-stage probability proportional-to-size sampling was used to select adults aged 20 years and older for the survey. The data came from 358 township and districts in Taiwan and was divided into seven strata, according to geographic location and degree of urbanization. The sampling design randomly selected townships and districts, *li*s (a *li* is a neighborhood-level unit created by the Taiwan Census Bureau), and individuals by probability proportional to their size. Interpersonal interviews were conducted using a structured questionnaire. The total sample size was 2,005, with a response rate of 53%. This study included 1,745 respondents, with no missing data for any of the study variables. The attrition analysis showed that respondents excluded due to missing data were more likely to be female, elderly, have a lower level of education, come from the lowest or the highest income levels, live in urban areas, and have poor intentions of conducting health-protective behaviors. We further assessed attrition by conducting multivariate analyses using social capital, gender, age, and education as the explanatory variables. We found that there was no difference in the pattern of the relationships between social capital and outcomes using the sample of 2,005 respondents and 1,745 respondents. Written informed consent was obtained from each respondent. The ethics committees/IRBs of the Academia Sinica of Taiwan approved this study and the consent procedure.

### Measurements

#### Outcome variables

The study sought to measure the participants’ intention to perform three behavioral outcome variables during a possible influenza epidemic: receiving a vaccine, wearing a mask, and washing their hands. Participants responded to a version of the following question for each of the three behavioral intention variables: “When a new type of influenza epidemic occurs in Taiwan, would you take the following actions [receive a flu shot, wear a face mask, wash your hands more frequently] to prevent flu transmission?”, based on a 5-point scale. The scale was recategorized into two groups: 1 (definitely yes, probably yes, neither yes nor no), and 0 (probably no, definitely no).

#### Explanatory variables

This study used two variables to represent the aspect of neighborhood support in the concept of bonding social capital. The first variable measured the number of neighbors with whom the respondent was on greeting terms and was recategorized into the following number categories: 0, 1–4, 5–9, 10–29, and ≥ 30, which were given scores of 1–5. The second variable measured the number of neighbors from whom the respondent could ask a favor when needed, such as receiving a mail delivery and taking care of or picking up children, and was recategorized into the following categories: 0, 1–2, 3–4, 5–9, and ≥ 10, which were given scores of 1–5. A composite score was created by averaging these two variables, with higher scores representing higher levels of neighborhood support (r = .59). Bridging social capital was measured by asking respondents to indicate membership in any associations (Yes vs. No). Linking social capital involved two dimensions: “general government trust” and “trust in the government’s capacity to handle an influenza epidemic”. General government trust was measured by asking the respondents to assign separate ratings to their central government, local government (county or municipal), and township (town, city, district) administrative offices regarding how much they trusted these government institutions, based on a 5-point scale. A composite score was created by averaging these three variables, with higher scores representing higher levels of general government support (α = .74). If some missing values were found on certain items, the mean value for the remaining items were used for the missing value. The concept of trust in the government’s capacity to handle an influenza pandemic was measured according to participant responses to the following three questions, based on a 5-point scale. Respondents evaluated whether the government fully informs the public with information regarding new types of influenza, whether they worry that the government might hide information about a new type of influenza, and whether they think that the government has the ability to manage an epidemic immediately if a new type of influenza occurs in Taiwan. A composite score was created by averaging these three variables, with higher scores representing a higher level of trust in the government’s ability to address an epidemic crisis (α = .53). If some missing values were found on certain items, the mean value for the remaining items were used for the missing value.

This study examined construct validity through an exploratory factor analysis on all of the social-capital variables. This analysis showed that each variable fits well under presumed dimensions and that there are significant relationships existing between the variables and the concepts. Many variables were also found to have significant relationships with the theoretical concepts of previous studies and, thus, to have construct validity. The variables on membership of organizations were positively correlated with self-rated health [26]. The variables regarding contacts with neighbors and government trust were positively related to individual health and status-based sociable resources (i.e., income) [27,28].

#### Control variables

This study controlled for two risk perception variables. Perceived susceptibility was measured based on “How likely do you think you will get infected with a new type of influenza?” Perceived severity was measured according to “How serious do you think it is to get infected with a new type of influenza?” These two variables were measured on a 5-point scale and were recategorized into two groups: high vs. low. The risk perception variables were suggested to be positively associated with health behavioral intention, based on the theory of the Health Belief Model [5]. Education was grouped into “less than high school,” “some college,” and “college graduate.” Monthly household income was categorized into five groups: “< NT$50,000,” “NT$50,000–89,999,” “NT$90,000–179,999”, “≥ NT$180,000” (US$1 = NT$32), and “missing”. Gender, age (20–34, 35–49, 50–54, ≥ 65), marital status (married vs. others), and locality (urban, suburban, rural) were suggested to be associated with either social capital or behavioral intent in prior studies and, thus, were included as control variables. Self-rated health was included as another control variable in order to rule out the potential for a confounding effect from a person’s health status in the relationship between social capital and behavioral intent. This variable was recategorized into two groups: 1 (very good, good, fair), and 0 (poor, very poor).

### Analysis

This study conducted a series of binary logistic regressions in the analyses. Two sets of binary logistic regressions models were used for assessing the unadjusted bivariate associations between each explanatory variable and outcome variable, as well as for adjusting the multivariate associations for sociodemographic and risk perception variables. Analyses were conducted separately according to type of behavioral intention. Assessing the variance inflation factor and tolerance score showed no multicollinearity problem among the independent variables in the regression models.

## Results

Table 1 shows the descriptive statistics and the bivariate analyses for the study variables. More than half of the respondents were male (52.5%) and married (59.6%), with 30.8% in the 20–34 age group. Nearly half of the respondents had a monthly household income of < NT$90,000 (52.2%), were college graduates (48.4%), and lived in urban areas (49.4%); 38.7% rated themselves as having poor health. Although 17.8% of the respondents perceived that they were susceptible to contracting a new type of influenza, 88.6% perceived being infected by this disease as serious. Most of the respondents reported that they intended to receive vaccination (78.8%), wear a mask (91.6%), and wash their hands more frequently (94.3%) should there be an influenza pandemic; 41% were members of at least one association. The mean scores were 3.03, 3.29, and 2.77 on a 5-point scale, respectively, for neighborhood support, general government trust, and trust in the government’s capacity to address an influenza pandemic. For the bivariate analyses, respondents who were male, had more income, had higher education, perceived high susceptibility to or severity of the disease, had higher neighborhood support, and had higher general government trust were more likely to intend to receive vaccination than their counterparts. Women who were younger, had higher family income, had a higher level of education, lived in urban areas, perceived higher susceptibility to or severity of the disease, were members of any association, and had higher general government trust were more likely to have the intention to wear a face mask than their counterparts. For the intention of washing their hands, respondents who were middle aged, married, lived in urban or rural areas, perceived high susceptibility to or severity of the disease, and had a higher level of neighborhood support or general government trust were likely to wash their hands more frequently than their counterparts.

**Table 1:** Descriptive statistics for the study variables and chi-squared tests for the intention to engage in health-protective behaviors.

Variables	Total (%)	Vaccination (%)	Wear a face mask (%)	Wash hands (%)
**Total**	*N* = 1745	78.80	91.63	94.38
***Sociodemogarphic factors and risk perception***				
**Gender**				
Male	52.49	81.77*	90.17*	93.78
Female	47.51	75.51	93.24	95.05
**Age**				
20–34	30.83	82.53	94.42*	91.82*
35–49	26.30	76.91	93.46	95.86
50–64	27.97	77.46	92.42	96.52
≥ 65	14.90	76.92	81.15	93.08
**Monthly household income**				
< NT$50,000	24.76	71.53*	87.96*	93.98
NT$50,000–89,999	27.39	80.96	93.31	94.56
NT$90,000–179,999	20.92	81.92	94.79	94.79
≥ NT$180,000	21.95	79.11	89.56	93.47
Missing	4.99	88.51	96.55	97.70
**Education**				
High school graduates	24.81	71.36*	82.91*	93.07
Some college	26.76	79.87	91.65	95.29
College graduates	48.42	82.01	96.09	94.56
**Marital status**				
Others	40.40	78.87	91.06	92.77*
Married	59.60	78.75	92.02	95.48
**Locality**				
Urban	49.40	80.05	93.16*	95.71*
Suburban	38.40	76.57	90.90	92.24
Rural	12.21	80.75	87.79	95.77
**Self-rated health**				
Poor	38.68	79.41	91.70	94.52
Good	61.32	78.41	91.59	94.30
**Perceived susceptibility**				
Low	82.23	77.56*	90.45*	93.66*
High	17.77	84.52	97.10	97.74
**Perceived severity**				
Low	11.40	65.33*	83.92*	90.95*
High	88.60	80.53	92.63	94.83
***Bonding social capital***				
**Neighborhood support** ^**a**^				
Low (< 2.1)	27.62	75.31*	90.46	91.70*
Middle (2.1–3.4)	32.78	77.62	91.78	94.23
High (> 3.4)	39.60	82.20	92.33	96.38
***Bridging social capital***				
**Association membership**				
No	59.03	77.96	90.19*	93.98
Yes	40.97	80.00	93.71	94.97
***Linking social capital***				
**General government trust** ^**a**^				
Low (< 3)	27.79	72.78*	88.25*	92.78*
Middle (3–3.9)	31.81	77.48	92.25	93.33
High (> 3.9)	40.40	83.97	93.48	96.31
**Trust in government’s capacity to handle an influenza pandemic** ^**a**^				
Low (< 2.6)	38.17	77.33	90.09	95.35
Middle (2.6–3.3)	26.02	79.30	92.51	92.73
High (> 3.3)	35.82	80.00	92.64	94.56

^**a**^The categories were based on tertiles.

*p <. 05.

Table 2 lists the binary logistic regression modeling results for the intention to receive vaccination. The results in the adjusted model revealed that male respondents were more likely than women to have the intention to receive vaccination (OR = 1.41). Compared with the respondents at the lowest income level, those at other income levels were more likely to have the intention to receive vaccination. Higher-educated respondents (OR = 1.64 for “some college” and OR = 1.62 for “college graduates”) were more likely to have the intention to receive vaccination. Respondents who perceived a high susceptibility (OR = 1.44) and a high severity of the disease (OR = 2.29) were more likely to have the intention to receive vaccination. Regarding the influence of social capital, both bonding social capital measured according to neighborhood support (OR = 1.19) and linking social capital measured based on general government trust (OR = 1.35) were consistently associated with the behavioral intention to receive vaccination, in both the unadjusted and adjusted models.

**Table 2:** Association between social capital, sociodemographic factors, risk perception, and the intention to receive vaccination.

Variables	OR (95% CI)^a^	AOR (95% CI)^b^
***Sociodemographic factors risk perception***		
**Gender**		
Female	1	1
Male	1.45 (1.16–1.83)**	1.41 (1.11–1.80)**
**Age**		
20–34	1	1
35–49	0.71 (0.52–0.96)*	0.70 (0.49–1.01)
50–64	0.73 (0.54–0.99)*	0.76 (0.50–1.13)
≥ 65	0.71 (0.54–1.02)	0.93 (0.58–1.51)
**Monthly household income**		
< NT$50,000	1	1
NT$50,000–89,999	1.69 (1.24–2.31)**	1.50 (1.07–2.11)*
NT$90,000–179,999	1.80 (1.29–2.53)**	1.60 (1.09–2.34)*
≥ NT$180,000	1.51 (1.09–2.08)*	1.40 (1.00–1.97)*
Missing	3.07 (1.54–6.12)**	2.50 (1.21–5.16)*
**Education**		
High school graduates	1	1
Some college	1.59 (1.17–2.17)**	1.64 (1.15–2.35)**
College graduates	1.83 (1.39–2.40)**	1.62 (1.19–2.34)*
**Marital status**		
Others	1	1
Married	0.99 (0.79–1.26)	1.12 (0.84–1.50)
**Locality**		
Urban	1	1
Suburban	0.82 (0.64–1.04)	0.86 (0.66–1.12)
Rural	1.05 (0.72–1.53)	1.18 (0.79–1.77)
**Self-rated health**		
Poor	1	1
Good	0.94 (0.74–1.19)	0.79 (0.61–1.01)
**Perceived susceptibility**		
Low	1	1
High	1.58 (1.13–2.20)**	1.44 (1.02–2.03)*
**Perceived severity**		
Low	1	1
High	2.20 (1.60–3.02)**	2.29 (1.63–3.21)**
***Bonding social capital***		
**Neighborhood support**	1.17 (1.05–1.31)**	1.19 (1.05–1.34)**
***Bridging social capital***		
**Association membership**		
No	1	1
Yes	1.13 (0.89–1.43)	1.10 (0.85–1.41)
***Linking social capital***		
**General government trust**	1.39 (1.21–1.60)**	1.35 (1.16–1.57)**
**Trust in government’s capacity to handle an influenza pandemic**	1.09 (0.94–1.25)	0.98 (0.84–1.15)

*p <. 05.

**p <. 01.

^**a**^Crude Odds Ratios.

^**b**^Adjusted Odds Ratios controlling all of the other variables.

Table 3 shows the results for the intention to wear a face mask. In the adjusted model, some variables (e.g., household income and locality) were statistically significant in the crude model but became nonsignificant in the adjusted model. The results in the adjusted model indicated that men (OR = .53) and respondents aged 65 and older (OR = .38) were less likely to have the intention to wear a face mask when they encountered a potential influenza pandemic. By contrast, higher-educated respondents (OR = 1.79 for “some college” and OR = 4.04 for “college graduates”), married respondents (OR = 1.55), and respondents who perceived higher susceptibility to and severity of the disease were more likely to wear a face mask than their counterparts. Regarding the relationship with social capital, respondents who were members of any association (OR = 1.65) and respondents who had higher general government trust (OR = 1.33) were more likely to have the intention to use a face mask.

**Table 3:** Association between social capital, sociodemographic factors, risk perception, and the intention to wear a face mask.

Variables	OR (95% CI)^a^	AOR (95% CI)^b^
***Sociodemographic factors and risk perception***		
**Gender**		
Female	1	1
Male	0.67 (0.47–0.94)*	0.53 (0.36–0.78)**
**Age**		
20–34	1	1
35–49	0.84 (0.50–1.42)	0.77 (0.43–1.40)
50–64	0.72 (0.44–1.18)	0.82 (0.43–1.60)
≥ 65	0.25 (0.16–0.41)**	0.38 (0.19–0.74)**
**Monthly household income**		
< NT$50,000	1	1
NT$50,000–89,999	1.91 (1.20–3.03)**	1.07 (0.63–1.77)
NT$90,000–179,999	2.49 (1.45–4.30)**	1.21 (0.65–2.23)
≥ NT$180,000	1.17 (0.76–1.82)	0.90 (0.56–1.45)
Missing	3.83 (1.17–12.56)*	1.63 (0.47–5.67)
**Education**		
High school graduates	1	1
Some college	2.26 (1.50–3.42)**	1.79 (1.09–2.93)**
College graduates	5.07 (3.30–7.79)**	4.04 (2.29–7.12)**
**Marital status**		
Others	1	1
Married	1.13 (0.80–1.59)	1.55 (1.01–2.36)*
**Locality**		
Urban	1	1
Suburban	0.73 (0.51–1.07)	0.75 (0.50–1.13)
Rural	0.53 (0.32–0.86)*	0.64 (0.38–1.12)
**Self-rated health**		
Poor	1	1
Good	0.99 (0.70–1.40)	0.73 (0.50–1.07)
**Perceived susceptibility**		
Low	1	1
High	3.53 (1.78–7.01)**	3.07 (1.51–6.21)**
**Perceived severity**		
Low	1	1
High	2.41 (1.58–3.68)**	2.27 (1.42–3.64)**
***Bonding social capital***		
**Neighborhood support**	1.12 (0.95–1.31)	1.22 (1.02–1.45)*
***Bridging social capital***		
**Association membership**		
No	1	1
Yes	1.62 (1.12–2.33)**	1.65 (1.11–2.45)*
***Linking social capital***		
**General government trust**	1.42 (1.16–1.74)**	1.33 (1.06–1.68)*
**Trust in government’s capacity to handle an influenza pandemic**	1.21 (0.98–1.50)	1.16 (0.92–1.47)

*p <. 05.

**p <. 01.

^**a**^Crude Odds Ratios.

^**b**^Adjusted Odds Ratios controlling all of the other variables.

The results for the intention to wash one’s hands more frequently during an influenza pandemic are shown in Table 4. The adjusted model indicates that respondents in suburban areas were less likely than those in urban areas to wash their hands frequently (OR = .54). Regarding the respondents’ risk perception, respondents who perceived high susceptibility to the disease were more likely to wash their hands (OR = 2.93). Similar to the results in Table 2, neighborhood support (OR = 1.43) and general government trust (OR = 1.45) were associated with washing one’s hands more frequently during an influenza pandemic.

**Table 4:** Association between social capital, sociodemographic factors, risk perception, and the intention to wash hands more frequently.

Variables	OR (95% CI)^a^	AOR (95% CI)^b^
***Sociodemographic factors and risk perception***		
**Gender**		
Female	1	1
Male	0.78 (0.52–1.19)	0.75 (0.49–1.16)
**Age**		
20–34	1	1
35–49	2.06 (1.19–3.59)**	1.92 (1.01–3.65)
50–64	2.47 (1.39–4.38)**	2.34 (1.11–4.92)
≥ 65	1.20 (0.68–2.12)	1.35 (0.61–3.01)
**Monthly family income**		
< NT$50,000	1	1
NT$50,000–89,999	1.11 (0.64–1.95)	0.95 (0.52–1.75)
NT$90,000–179,999	1.17 (0.63–2.14)	0.99 (0.51–1.94)
≥ NT$180,000	0.92 (0.52–1.62)	0.87 (0.48–1.58)
Missing	2.72 (0.63–11.67)	2.66 (0.59–12.02)
**Education**		
High school graduates	1	1
Some college	1.51 (0.86–2.65)	1.72 (0.88–3.37)
College graduates	1.29 (0.80–2.08)	1.70 (0.88–3.28)
**Marital status**		
Others	1	1
Married	1.65 (1.09–2.48)*	1.18 (0.71–1.97)
**Locality**		
Urban	1	1
Suburban	0.53 (0.35–0.82)*	0.54 (0.34–0.84)*
Rural	1.02 (0.48–2.14)	0.98 (0.45–2.12)
**Self-rated health**		
Poor	1	1
Good	0.96 (0.63–1.46)	0.90 (0.58–1.41)
**Perceived susceptibility**		
Low	1	1
High	2.93 (1.35–6.39)*	2.93 (1.33–6.45)**
**Perceived severity**		
Low	1	1
High	1.82 (1.07–3.11)*	1.62 (0.92–2.85)
***Bonding social capital***		
**Neighborhood support**	1.50 (1.22–1.83)**	1.43 (1.15–1.78)**
***Bridging social capital***		
**Association membership**		
No	1	1
Yes	1.21 (0.79–1.84)	0.97 (0.62–1.52)
***Linking Social Capital***		
**General government trust**	1.45 (1.13–1.83)**	1.45 (1.11–1.89)**
**Trust in government’s capacity to handle an influenza pandemic**	0.92 (0.72–1.20)	0.83 (0.63–1.11)

*p <. 05.

**p <. 01.

^**a**^Crude Odds Ratios.

^**b**^Adjusted Odds Ratios controlling all of the other variables.

## Discussion

Based on the typology of Szreter and Woolcock (2004), this study examined different forms of social capital and their relationships with behavioral intention in response to an influenza pandemic [19]. Regarding linking social capital, this study found that only general government trust was associated with behavioral intention. By contrast, whether or not respondents trusted the government’s capacity to manage the epidemic was not associated with any intention variables. Possible explanations may come from previous research on trust and risk management. Szreter and Woolcock (2004) have identified two dimensions of trust: the first dimension is based on the relationship between the trusting person and the other (relational trust), and the second dimension is based on past behavior of the other (calculative trust) [29,30]. They have suggested that relational trust is a more powerful predictor of compliance with recommended behaviors than calculative trust, particularly in an unknown situation. Because of the high degree of uncertainty surrounding a new type of influenza, people typically do not demonstrate the ability to fully process messages from the government [31]. People must make quick judgments, based on emotion and a general feeling toward the government, in taking action. This suggests that long-standing government trust should be cultivated by a government with its citizens, long before a disease epidemic occurs.

Compared with the significant relationship between bridging social capital (membership association) and the intention to wear a face mask, this study found that bonding social capital in a neighborhood was associated with all types of behavioral intention. Neighborhood-level social capital may facilitate greater interaction among neighbors and allow higher health information flow, which might be crucial for promoting health practices during an influenza pandemic [8]. Bonding social capital can effectively provide community resources in epidemic emergencies by mobilizing local institutions for action and by providing information and awareness about the disease through social networks, as well as by promoting discussion and problem solving regarding feasible actions [32]. In addition, peer pressure or moral responsibility, as derived from the solidarity of local social networks, may drive people to implement health measures to prevent the spread of the disease. Some researchers have suggested that increasing community-level social capital is especially recommended in disaster preparedness because it can help to build the social infrastructure necessary for a community to prevent and respond to outbreaks of infectious diseases [33].

Consistent with prior studies, the results of this study showed that people perceived low susceptibility to contracting the disease (17.8%) but a high severity of disease (88.6%) during a possible, future influenza pandemic [34]. This study found that perceived severity of the disease was associated with the intention to receive vaccination and wear a face mask but was not associated with the intention to wash hands more frequently. It seems that the higher the perceived severity of the disease, the higher the cost (i.e., expense of receiving a flu shot) the person is willing to pay to implement a health measure. In comparison with the different types of behavioral intention, the results suggested that respondents with higher education demonstrated a higher intention than did lower-educated respondents to receive vaccination and wear a face mask. In addition, males and higher-income people were more likely to have the intention to receive vaccination, which was consistent with previous studies [35]; however, these relationships did not apply to the intention of washing their hands and wearing a face mask. These findings may be related to gender differences regarding the belief of the effectiveness of vaccination and the perceived expense of receiving a new type of flu shot [36].

The findings of this study should be considered in the context of certain limitations. First, this study performed no longitudinal social capital measurements; therefore, the relationships found between social capital and the outcome should perhaps be more cautiously interpreted as mere associations. Second, this study did not measure all aspects of social capital (i.e., reciprocity) and this study did not measure bridging social capital directly, such as by asking the respondents whether they had an ethnically diverse social network. Nevertheless, association memberships have been proposed to represent bridging social capital and to indicate interpersonal relationships outside the circle of family and friends. Third, although the appropriate level for analyzing social capital is still disputed, this study only assessed social capital as an individual-level attribute [37]. Arguably, this approach could lead to difficulty in distinguishing this concept from social support [38]. However, many studies have assessed individual-level social capital and showed its relationship to one’s health status [37]. Fourth, this study relied on self-reporting respondents; therefore the findings may be subject to social desirability and recall bias [12]. Last, the generalizability of this study may be limited because of the low response rate (53%). It is now understood that response rates for survey research have been declining in many countries for the past decades [39]. These declining rates have led to concerns that nonresponse error may bias survey estimates. Taiwan Social Change Survey has estimated that about 20–25% of the non-response rates were due to the discrepancy of registered addresses and actual living addresses [25]. Studies have suggested through updating addresses from other supplemental sampling frames, such as postal addresses or other commercially available databases, might remedy the problem of low-response rate in a household survey [40].

## Conclusion

Despite the limitations, the findings of this study provide a recommendation for policymakers to promote bonding social capital and linking social capital as crucial for resolving health emergencies and other situations that place the citizenry at risk. The study results indicate the critical role that local, interpersonal networks play in enhancing communication platforms and in augmenting government credibility if a country is to mount a viable defense against a future influenza pandemic.
